# Characterization of Cellular and Acellular Analytes from Pre-Cystectomy Liquid Biopsies in Patients Newly Diagnosed with Primary Bladder Cancer

**DOI:** 10.3390/cancers14030758

**Published:** 2022-02-01

**Authors:** Stephanie N. Shishido, Salmaan Sayeed, George Courcoubetis, Hooman Djaladat, Gus Miranda, Kenneth J. Pienta, Jorge Nieva, Donna E. Hansel, Mihir Desai, Inderbir S. Gill, Peter Kuhn, Jeremy Mason

**Affiliations:** 1 Convergent Science Institute in Cancer, Michelson Center for Convergent Bioscience, University of Southern California, Los Angeles, CA 90089, USA; sshishid@usc.edu (S.N.S.); salmaans@usc.edu (S.S.); courcoub@usc.edu (G.C.); 2 Catherine & Joseph Aresty Department of Urology, Institute of Urology, Keck School of Medicine, University of Southern California, Los Angeles, CA 90033, USA; djaladat@med.usc.edu (H.D.); gmiranda@med.usc.edu (G.M.); mihir.desai@med.usc.edu (M.D.); igill@med.usc.edu (I.S.G.); 3 The James Buchanan Brady Urological Institute, Johns Hopkins University School of Medicine, Baltimore, MD 21231, USA; kpienta1@jhmi.edu; 4 Norris Comprehensive Cancer Center, Keck School of Medicine, University of Southern California, Los Angeles, CA 90033, USA; jorge.nieva@med.usc.edu; 5 Department of Pathology, School of Medicine, University of California San Diego, La Jolla, CA 92093, USA; hansel@ohsu.edu; 6 Department of Biomedical Engineering, Viterbi School of Engineering, University of Southern California, Los Angeles, CA 90089, USA; 7 Department of Aerospace and Mechanical Engineering, Viterbi School of Engineering, University of Southern California, Los Angeles, CA 90089, USA; 8 Department of Biological Sciences, Dornsife College of Letters, Arts, and Sciences, University of Southern California, Los Angeles, CA 90089, USA

**Keywords:** bladder cancer, urothelial carcinoma, cystectomy, liquid biopsy, HDSCA, circulating tumor cell, large extracellular vesicle, peripheral blood

## Abstract

**Simple Summary:**

The standard of care for patients diagnosed with localized bladder cancer (BCa) is cystectomy. However, emerging evidence shows that the patients receiving surgical intervention often experience a return of their cancer. Examining patient blood samples for rare events, such as circulating tumor cells (CTCs) and large extracellular vesicles (LEVs), may reveal biomarkers indicative of the presence of cancer and provide an insight into disease progression. Through computational methodologies, each event in the blood was characterized to determine its rarity within the sample and then compared to events found in normal donors. We demonstrate that a wide range of CTCs and LEVs are found in significantly higher proportions among BCa patients compared to normal donors. This result suggests that the blood liquid biopsy is a proficient analyte for detecting BCa to ultimately guide clinical decisions to improve patient treatment outcomes.

**Abstract:**

Urinary bladder cancer (BCa) is the 10th most frequent cancer in the world, most commonly found among the elderly population, and becomes highly lethal once cells have spread from the primary tumor to surrounding tissues and distant organs. Cystectomy, alone or with other treatments, is used to treat most BCa patients, as it offers the best chance of cure. However, even with curative intent, 29% of patients experience relapse of the cancer, 50% of which occur within the first year of surgery. This study aims to use the liquid biopsy to noninvasively detect disease and discover prognostic markers for disease progression. Using the third generation high-definition single cell assay (HDSCA3.0), 50 bladder cancer patient samples and 50 normal donor (ND) samples were analyzed for circulating rare events in the peripheral blood (PB), including circulating tumor cells (CTCs) and large extracellular vesicles (LEVs). Here, we show that (i) CTCs and LEVs are detected in the PB of BCa patients prior to cystectomy, (ii) there is a high heterogeneity of CTCs, and (iii) liquid biopsy analytes correlate with clinical data elements. We observed a significant difference in the incidence of rare cells and LEVs between BCa and ND samples (median of 74.61 cells/mL and 30.91 LEVs/mL vs. 34.46 cells/mL and 3.34 LEVs/mL, respectively). Furthermore, using classification models for the liquid biopsy data, we achieved a sensitivity of 78% and specificity of 92% for the identification of BCa patient samples. Taken together, these data support the clinical utility of the liquid biopsy in detecting BCa, as well as the potential for predicting cancer recurrence and survival post-cystectomy to better inform treatment decisions in BCa care.

## 1. Introduction

Bladder cancer (BCa) is the tenth most common cancer in the world, representing 3% of all new cancer cases [1]. Urothelial carcinoma (~90%) is the most frequent BCa histology diagnosed in the U.S., and can be subdivided by stage, grade, and subtype (conventional or variant morphology) [2]. Less common types include squamous (2–5%), adenocarcinoma (2%), and neuroendocrine (1%), as well as other rare tumors (<1%). Tumors that are confined to the lamina propria of the bladder are termed non-muscle invasive BCa (NMIBC; Ta, Tis (carcinoma in situ), T1), while those that invade the muscularis propria are called muscle invasive BCa (MIBC, T2-T4), an advanced stage with life threatening consequences requiring surgical management. BCa is highly lethal once cells have spread from the primary tumor to surrounding tissues and distant organs [3]. Cystectomy, the surgical removal of the bladder, is used to treat most BCa patients, as it offers the best chance of cure. The procedure can be performed alone or in combination with other treatments and can be considered a first-line intervention in cases of superficial tumors with severe anaplasia.

We have previously reported on the clinically observed patterns of relapse following cystectomy. Metastases developed in 29% of patients (*n* = 812), resulting in a five-year overall survival rate of 20.4%, compared to 78.6% in those without relapse (*n* = 1983) [4,5]. Most metastatic progression occurs within the first 24 months. In another study, information theory and machine learning algorithms were employed to create predictive models around this BCa database, in which the primary predictors of recurrence and survival after radical cystectomy were determined to be pathologic T stage and subgrouping into localized or metastatic conditions [3]. Clinical T stage had a lower predictive signal than the true pathologic T stage. This loss of valuable information may especially affect those cases in which there is an underestimation of disease severity prior to surgery [6]. This recognizes the limitation of current clinical staging at the time of diagnosis and highlights the importance of precision cell and tissue analysis in differentiating patients by outcome prior to and following surgical intervention.

The early relapse in primary BCa patients undergoing cystectomy may be attributed to the presence of pre-existing subclinical metastatic disease in these patients [4]. Current prominent methods for detection, diagnosis, and surveillance of the disease are based on urine cytology and cystoscopy. Urine cytology, while non-invasive, approximately yields a low sensitivity of 38% and a specificity of 98% [7]. On the other hand, cystoscopy has a higher sensitivity, between 65% and 90% depending on the subtype, but is a highly invasive procedure with significant inter- and intra-observer variation in tumor stage and grade [8]. Thus, there is great need for improving the current clinical paradigm of diagnostic workup and treatment planning. We hypothesized that the liquid biopsy as a biomarker of systemic disease may be diagnostic of subclinical metastatic disease and prognostic of early relapse. If proven correct, it could serve as a surrogate marker to guide the addition or use of alternative therapy as opposed to surgical intervention alone in patients diagnosed with BCa. A comprehensive analysis of the blood-based liquid biopsy may assist in solving complex clinical problems by tracking cellular evolution and phenotypic populations, revealing treatments that are not efficacious for specific patients, thus developing a stratification system in order to avoid unnecessary surgical intervention.

Circulating tumor cells (CTCs) shed by the tumor are often detectable in the peripheral blood (PB) of cancer patients and have been associated with poor prognosis and early relapse [8,9,10,11]. Busetto et al., observed a strong correlation between the detection of CTCs by CellSearch^®^ and the time to first recurrence [9]. Furthermore, in a meta-analysis of 2161 BCa patients from 30 published articles, Zhang et al., showed that the number of CTCs detected in the PB correlated with tumor stage, histological grade, metastasis, and regional lymph node metastasis [10]. These studies indicate that the presence of CTCs in the PB is an independent predictive indicator of poor outcomes for BCa patients. The work presented here is based on a third-generation comprehensive liquid biopsy [12]. This non-enrichment based, high-content direct imaging methodology is capable of providing both the visualization and characterization of a broad range of CTCs that are present in circulation, along with molecular parameters (DNA and protein) at both the cellular and acellular (large extracellular vesicles (LEVs) and cell-free DNA (cfDNA)) levels. We have previously reported the value of single-cell genomic analysis conducted on this platform showing compatibility with clinical practice [12,13,14].

The third generation high-definition single cell assay (HDSCA3.0) liquid biopsy workflow [15,16,17] was designed for rare cell identification with immunocytochemistry [18] along with downstream molecular characterization in order to deliver diagnostic pathology-quality data for clinical decision making [13,14,19,20,21]. The primary objective of the present study was to investigate the prognostic significance of CTCs in BCa patients from PB samples taken prior to cystectomy. The secondary objective was to assess the association between CTC presence and known clinical data metrics, such as clinical or pathological staging and histological subtype. This study aims at establishing evidence for the clinical utility of the liquid biopsy in BCa with the future goal of predicting metastatic relapse post-cystectomy and enable clinical interventions that can lead to improved outcomes.

## 2. Materials and Methods

### 2.1. Study Design

This was a multiple institution prospective study of patients diagnosed with BCa in which PB samples were collected before cystectomy and prior to any procedures. Eligible patients underwent cystectomy for surgical removal of the primary tumor from the bladder. University of Southern California’s Keck School of Medicine (Keck; *n* = 25) samples were collected between January and November 2020. Samples from the University of California San Diego (UCSD; *n* = 9), Johns Hopkins Hospital (JHH; *n* = 13), and LAC/USC Medical Center (LAC; *n* = 3) were collected between January 2016 and November 2017. The Keck patient subset has prospectively collected clinical, radiologic, and pathologic data elements as well as a limited amount of follow-up data. For this cohort, recurrence is defined as any clinical recurrence majority shown radiologically, either symptomatic or not. Patient recruitment took place according to an institutional review board approved protocol at each site, and all study participants provided written informed consent. Here, we present the liquid biopsy analysis from a total of 50 BCa patients. Additionally, 50 normal donor (ND) samples from individuals with no known pathology were provided from Epic Sciences (San Diego, CA, USA).

### 2.2. Blood Sample Processing

PB samples were collected in 10 mL blood collection tubes (Cell-free DNA, Streck, La Vista, NE, USA) and processed by the Convergent Science Institute in Cancer (CSI-Cancer) at the University of Southern California within 24–48 h as previously described [18]. Briefly, samples underwent red blood cell lysis, followed by plating the entire nucleated cell fraction on custom glass slides (Marienfeld, Lauda, Baden-Württemberg, Germany) at approximately 3 million cells per slide prior to long-term cryostorage at −80 °C and rare cell analysis.

### 2.3. Blood Sample Staining and Imaging

For HDSCA analysis, each test consisted of two slides generated from the PB sample for an average of 0.74 mL blood analyzed. Slides were processed at room temperature using the IntelliPATH FLX™ autostainer (Biocare Medical LLC, Irvine, CA, USA) as previously described [12]. Briefly, samples were stained with 2.5 ug/mL of a mouse IgG1 anti-human CD31:Alexa Fluor^®^ 647 mAb (clone: WM59, MCA1738A647, BioRad, Hercules, CA, USA) and 100 ug/mL of a goat anti-mouse IgG monoclonal Fab fragments (115-007-003, Jackson ImmunoResearch, West Grove, PA, USA), permeabilized using 100% cold methanol, followed by an antibody cocktail consisting of mouse IgG1/Ig2a anti-human cytokeratins (CKs) 1, 4, 5, 6, 8, 10, 13, 18, and 19 (clones: C-11, PCK-26, CY-90, KS-1A3, M20, A53-B/A2, C2562, Sigma, St. Louis, MO, USA), mouse IgG1 anti-human CK 19 (clone: RCK108, GA61561-2, Dako, Carpinteria, CA, USA), mouse anti-human CD45:Alexa Fluor^®^ 647 (clone: F10-89-4, MCA87A647, AbD Serotec, Raleigh, NC, USA), and rabbit IgG anti-human vimentin (Vim) (clone: D21H3, 9854BC, Cell Signaling, Danvers, MA, USA). Lastly, slides were incubated with Alexa Fluor^®^ 555 goat anti-mouse IgG1 antibody (A21127, Invitrogen, Carlsbad, CA, USA) and 4′,6-diamidino-2-phenylindole (DAPI; D1306, ThermoFisher) prior to mounted with a glycerol-based aqueous mounting media. Samples were imaged using automated high-throughput fluorescence scanning microscopy at 10× objective magnification generating 2304 frames images per fluorescence channel per slide.

### 2.4. Rare Event Detection and Classification

As previously reported [12], rare cell candidates were detected using a custom computational methodology termed OCULAR (Outlier Clustering Unsupervised Learning Automated Report). Fluorescent images were used to segment each cell using the “EBImage” R package (EBImage_4.12.2) and extract 761 quantitative morphometric parameters based on the nuclear and cytoplasmic morphology and biomarker expression (CK, Vim, CD45/CD31) in a 4-channel immunofluorescence assay (DAPI, AlexaFluor^®^ 488, AlexaFluor^®^ 555, AlexaFluor^®^ 647). Additionally, the algorithm identified DAPI-negative CK-positive events into a separate report to be classified as large extracellular vesicle (LEV) candidates [20].

Manual reporting was conducted on the identified events to check for signal intensity and distribution, as well as morphology. Images of candidate rare events were presented to a hematopathologist-trained technical analyst for analysis and interpretation. Rare events were classified into 12 categories (8 cellular, 4 LEV) based on the combination of immunofluorescent marker expression in the previously reported 4 channels. Epithelial-like CTCs (epi.CTCs) were classified as cells that were CK-positive, Vim-negative, and CD45/CD31-negative, with distinct appearing nucleus by DAPI morphology as previously described [12,18]. Epi.CTCs expressing Vim were classified as mesenchymal-like CTCs (mes.CTCs). White blood cell (WBC) counts of whole blood were determined automatically (Medonic M-series Hematology Analyzer, Clinical Diagnostic Solutions Inc., Fort Lauderdale, FL, USA) and the number of WBCs detected by the assay per slide was used to calculate the actual amount of blood analyzed per test so that results are presented as fractional values of events/mL.

LEV candidates were positive for CK with variable Vim and CD45/CD31 expression. LEVs were identified through the OCULAR methodology outlined above with careful identification for those that were either free-floating or in close proximity to cells. Due to the close proximity of the cell-attached LEVs, OCULAR interpreted both as a single cellular event. Manual classification to separate these two entities as individual rare events was employed to correct for the computational oversimplification of OCULAR. Further, corrections included excluding any halos, bubbles, or light refractions resembling the morphology of LEVs (round and membranous) when examining frames of patient samples through the CK channel. A maximum threshold of three LEVs per frame was used to rule out CK-positive junk particles that may have landed on the slide during processing.

### 2.5. Statistical Analysis

Statistical significance was determined at a *p*-value ≤ 0.05. To perform statistical analysis of the clinical, radiologic, and pathologic data, we used two statistical tests: Spearman’s rank correlation coefficient [22] and the Mann-Whitney U test, also known as the Wilcoxon rank sum test [23,24]. The Spearman rank test was used to calculate the correlation between continuous variables as we are not strictly evaluating the degree of linear relationship, but rather the degree of monotonic relationship between the two target variables. In addition, it was also non-exclusively applied to evaluate the correlation between continuous variables and categorical variables that have a well-defined ordinal encoding and multiple outcomes. For example, the clinical T stage encoded such that the available classifications (T0, Tis, Ta, T1, T2a, T3b, T4a) were assigned to ordinal values from 0 to 6. To evaluate the correlation between continuous and categorical data without a well-defined ordinal encoding, we also performed the Wilcoxon rank sum test.

The Wilcoxon rank sum test, which determines whether two samples are likely to derive from the same population, is appropriate for small datasets, and does not require that the data be paired or normally distributed [25]. This nonparametric test is calculated based on the ranks (or order) of the numerical variables, making it robust with respect to outliers. For categorical variables that can have more than two classifications, the Wilcoxon rank sum test is calculated between all possible classification pairs. For example, the correlation between total rare cell count vs. clinical predominant cancer cell type (Urothelial, Other, No Tumor) is calculated for all combinations: Urothelial vs. Other, Urothelial vs. No Tumor, and Other vs. No Tumor. All statistical tests were performed in Python (version 3.8.5) with the Scipy library (version 1.5.0).

To visualize the morphometrics of detected cellular events, a two-dimensional tSNE (t-distributed stochastic neighbor embedding) was used [26]. To aid the identification of clusters in the tSNE, a clustering algorithm was used. Specifically, we applied agglomerative clustering imported from the sklearn library version 0.23.2 [27]. For the clustering parameters, we used Ward linkage and a Euclidian distance metric [28].

### 2.6. Patient Level Classification Modeling

Classification models were used to test whether BCa patients can be discerned from NDs utilizing liquid biopsy data alone (i.e., whether one has distinct rare event populations when compared to the other). The python library sklearn version 0.23.2 was used to develop the machine learning models [27]. Two slides each from 50 ND samples were collected to mirror the 50 BCa patient samples. For each individual, the data utilized in the classification models were the counts for each cell and event classification per ml of blood averaged across both slides. Three different classification models (random forest (RF), support vector machine (SVM) and naive Bayes (NB)) were tested to produce a binary outcome indicating whether an individual is within the BCa or ND category. We employed a 5-fold cross validation method to test each model architecture in which the dataset was divided into five equal folds of 20 individuals. Each fold is then used as a test set for a model built with the remaining four, yielding five models for each or RF, SVM, and NB (i.e., 15 total models). We employed a grid search algorithm to find optimal hyperparameters for the RF and SVM. Final model metrics are averages across all models of the same type.

## 3. Results

A total of 50 patients with primary BCa were accrued for this study, each providing a single PB sample obtained prior to cystectomy. Site-specific liquid biopsy data are provided in Figure S1. Three patients within the Keck subset withdrew consent after surgery and are not included in the statistical analyses between liquid biopsy and clinical data. Clinical and demographic data metrics were collected for the Keck subset (*n* = 22) and are provided in Table 1. At the time of data collection, two of the patients had recurred and one was deceased. ND information was limited to participant age (median 57, range 45–82, mean 58.9).

### 3.1. Liquid Biopsy Analysis Prior to Cystectomy

A complete blood cell count was taken at CSI-Cancer prior to blood processing. For the 50 BCa samples included here, there was a median WBC count of 6.75 (range 3.3–25; mean 7.5) million cells/mL PB. For all BCa samples, total rare event (total cells and LEVs) detection had a median of 132.67 events/mL (range 38.11–1220.51; mean 230.33). For ND samples, total rare event detection had a median of 38.50 events/mL (range 4.39–141.55; mean 47.86). A significant difference was observed between the BCa patients and ND (*p*-value < 0.0001).

### 3.2. Rare Cell Characterization

**Figure 1:**
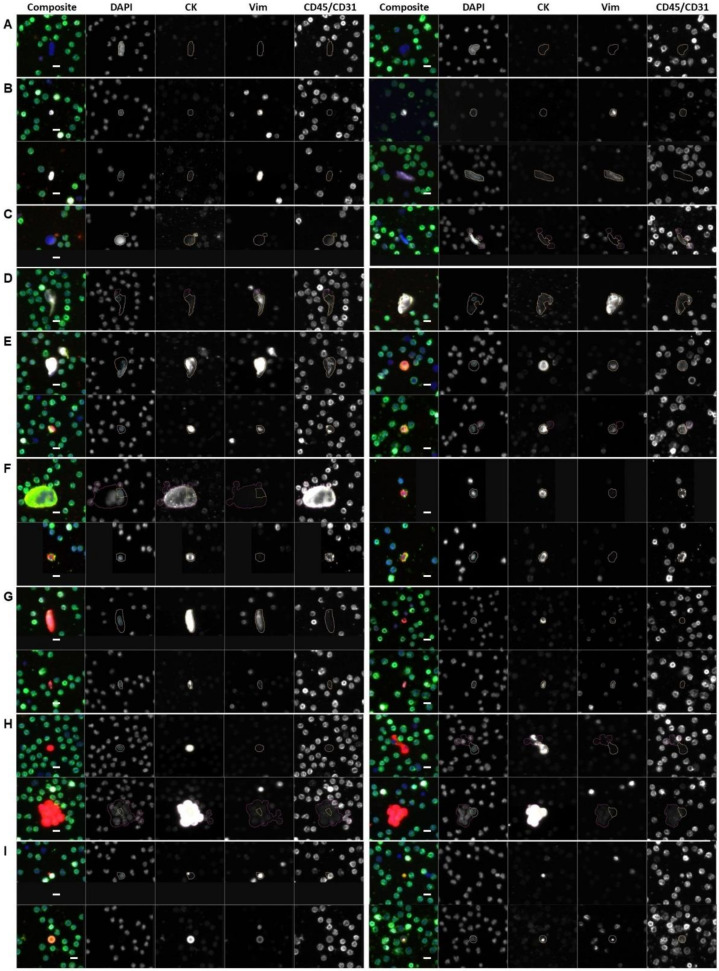
Gallery of representative rare events detected by HDSCA3.0 in PB samples collected from BCa patients prior to cystectomy or ND with no known pathology. (**A**–**H**) rare cells and (**I**) LEVs. (**A**) DAPI only; (**B**) Vim; (**C**) CD45/CD31; (**D**) Vim|CD45/CD31; (**E**) CK|Vim|CD45/CD31; (**F**) CK|CD45/CD31; (**G**) mes.CTC; (**H**) epi.CTC; (**I**) LEVs (top left: CK only; bottom left: CK|Vim|CD45/CD31; top right: CK|CD45/CD31; bottom right: CK|Vim.)] Blue: DAPI, Red: CK, White: Vim, Green: CD45/CD31. Images taken at 100× magnification. Scale bar = 10 µm.

**Figure 2:**
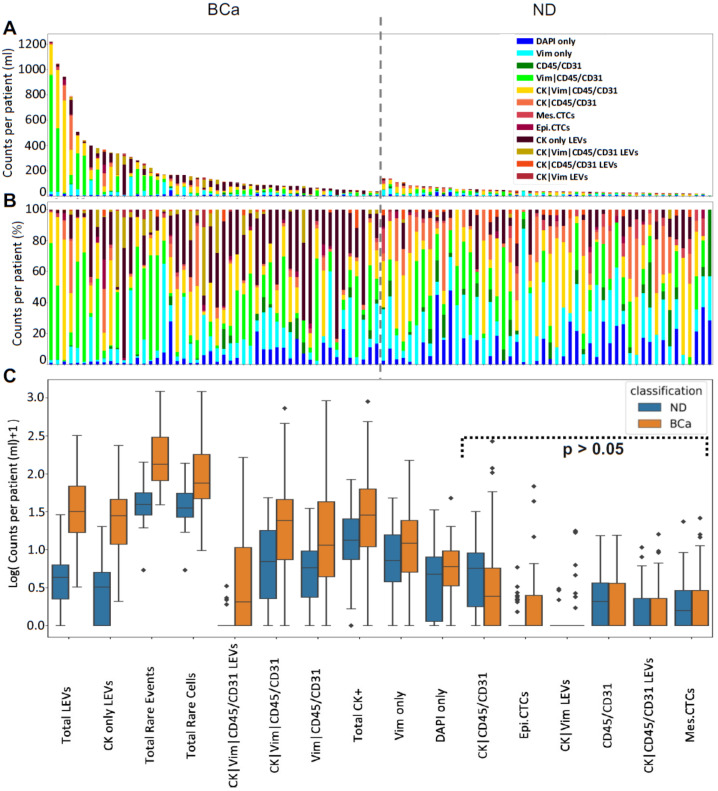
Rare event detection using HDSCA3.0 in PB samples collected from BCa patients prior to cystectomy and ND. (**A**) Enumeration and (**B**) frequency of each rare event by channel-type specification. (**C**) Graphical representation of the channel-type rare events/mL between BCa and ND samples ordered by degree of statistical significance. Symbols indicate outliers. Channel-type specifications that were not statistically significant across the two classifications are highlighted (*p* > 0.05).

We identified eight cellular categories defined by nuclear DAPI signal and rely on the expression of the different biomarkers in each channel. A gallery of CTCs and graphical representation of the frequency of each rare event identified per test for each patient sample are shown in Figure 1 and Figure 2. Total rare cell detection for the BCa samples had a median of 74.61 cells/mL (range 8.75–1213.69; mean 178.40). The ND samples presented with a median rare cell detection of 34.46 cells/mL (range 4.39–137.03; mean 43.21). A statistically significant difference in total rare cell detection was observed between the BCa patients and ND samples (*p*-value < 0.0001).

Total CK-positive cells were detected with a median of 27.59 cells/mL (range 0–895.72; mean 79.36) from all BCa samples. The ND samples had a median of 12.90 cells/mL (range 0–83.24; mean 18.96). There was a statistically significant difference in total CK-positive cell detection between BCa patient and ND samples (*p*-value = 0.0093). Only one BCa patient (2%) did not present with CK-positive cells at the time of sample collection. Using a threshold of positivity of >5 cells/mL, a total of 44 samples (88%) were positive for CK expressing cells. The frequency of CK-positive cells detected within the total rare cell population varied. Overall, there was a median frequency of 30.2% (range 0–97%; mean 36%) in the BCa samples.

Epi.CTCs were detected with a median of 0 cells/mL (range 0–27; mean 1.2) from BCa patient samples. Mes.CTCs were detected with a median of 0 cells/mL (range 0–25.12; mean 2.33) from BCa patient samples. There was no statistically significant difference in epi.CTCs/mL or mes.CTCs/mL observed between BCa patient and ND samples.

Additional candidate CTCs detected include CK|CD45/CD31 (median 1.44 cells/mL; range 0–267.84; mean 13.76) and CK|Vim|CD45/CD31 (median 23.19 cells/mL; range 0–729.44; mean 60.09). Other detectable rare cells include morphologically distinct Vim|CD45/CD31 (median 10.51 cells/mL; range 0–919.24; mean 68.36), CD45/CD31 only (median 0 cells/mL; range 0–14.49; mean 1.89), DAPI only (median 5.00 cells/mL; range 0–46.86; mean 6.76), and Vim only (median 11.18 cells/mL; range 0–149.57; mean 22.04). There was a statistically significant difference between BCa patient and ND samples in cellular enumeration of Vim|CD45/CD31 (*p*-value = 0.0018), CK|Vim|CD45/CD31 (*p*-value = 0.0003), Vim only (*p*-value = 0.0406), DAPI only (*p*-value = 0.0430). The biological significance of these cellular populations has not been determined.

The most prevalent cell types observed in the PB of BCa patients prior to cystectomy were Vim|CD45/CD31 (median 15.19%; range 0–80.53%; mean 28.64%) and CK|Vim|CD45/CD31 cells (median 22.99%; range 0–79.49%; mean 26.10%), followed by Vim only cells (median 14.02%; range 0–81.13%; mean 21.94%). Out of all the rare cells detected across patient samples, Vim|CD45/CD31 cells constituted 45.24%, CK|Vim|CD45/CD31 cells constituted 31.05% and Vim only cells constituted 10.74%. We identified a positive correlation between mes.CTC and CK|Vim|CD45/CD31 (spearman coefficient = 0.58, *p*-value < 0.001), as well as two other cellular categories (Vim only (spearman coefficient = 0.358, *p*-value = 0.01), CK|CD45/CD31 (spearman coefficient = 0.292, *p*-value = 0.040)). This suggests that the cellular populations are associated with each other and represent the heterogeneity of the disease.

**Figure 3:**
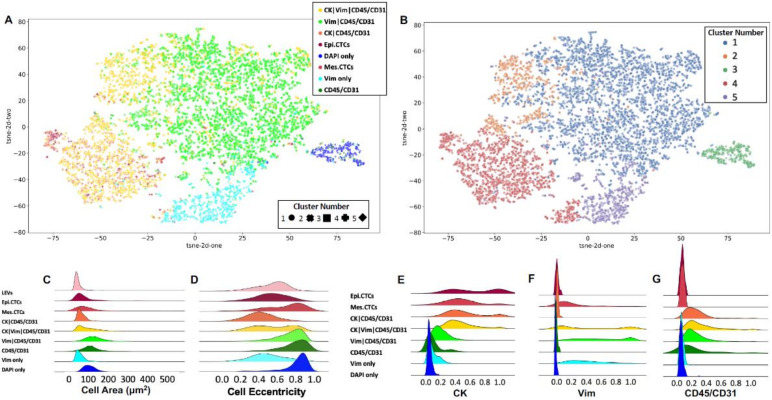
Morphometric analysis of individual events detected by HDSCA3.0 in PB samples collected from BCa patients prior to cystectomy. (**A**) tSNE plot of rare cellular events depicting the underlying morphological heterogeneity. Each point represents a single cell and is color coded according to its channel-type classification. (**B**) The same tSNE plot color coded according to a distinct cluster number, as determined by a clustering algorithm. The cells group in multiple clusters spanning across classifications. Visualization of the probability density distributions for select morphometric parameters across channel-type classifications: (**C**) cell area, (**D**) cell eccentricity, (**E**) median CK signal intensity, (**F**) median Vim signal intensity, (**G**) median CD45/CD31 signal intensity.

To visualize the cellular subgroups and their similarities with respect to morphometrics we used eight key measures. The first four are obtained from the median immunofluorescence intensity of DAPI, CK and CD45/CD31 channels. The second set of four are the area and eccentricity for the cell and the nucleus. The morphometrics were visualized by a two-dimensional tSNE plot shown in Figure 3. Each rare cell is represented with a single point, which is color coded based on its classification. Furthermore, to aid the interpretation of the cellular clusters, agglomerative clustering was applied to separate the cells in five clusters based on the same set of morphometrics. The plot markers were adjusted accordingly to match each cell to the corresponding cluster, as determined by the algorithm.

The channel-type classified cellular populations had observable morphological heterogeneity which is displayed in Figure 3. Morphological analysis indicates multiple distinct cellular populations independent from biomarker expression. The DAPI only and Vim only cells cluster distinctly from the other channel-type groups by their morphology, forming cluster numbers 3 and 5, respectively. The epi.CTC, mes.CTC, and CK|CD45/CD31 cells clustered together in cluster number 4, suggesting these are morphologically related. The CK|Vim|CD45/CD31 cell population has multiple distinct morphological subtypes, with a subset of cells that cluster with the epi.CTCs, mes.CTCs, and CK|CD45/CD31 cells. Another CK|Vim|CD45/CD31 subset is morphologically similar to the Vim|CD45/CD31 cells, which were strongly positively correlated (spearman coefficient = 0.40, *p*-value = 0.004). This suggests high heterogeneity of the CK|Vim|CD45/CD31 population, which may represent multiple distinct cellular phenotypes related to BCa.

### 3.3. LEV Detection

LEVs were classified by DAPI negativity, CK signal positivity and distribution, as well as morphology. Total LEV detection for the BCa patient samples had a median of 30.91 LEVs/mL (range 2.22–319.08; mean 51.92). The ND samples presented with a median of 3.34 LEVs/mL (range 0–27.91; mean 4.65), which was significantly lower than that detected in the BCa samples (*p*-value < 0.0001). In BCa patient samples, LEVs were detected either alone (*n* = 740; 44.6%) or in close proximity to cells (*n* = 918; 55.4%). In ND samples, these LEV populations totaled 85 (45.9%) and 100 (54.1%), respectively.

CK only LEVs were detected in all BCa patients with a median of 27.06 LEVs/mL (range 1.08–235.92; mean 37.80). CK|Vim|CD45/CD31 LEVs were also detected in 27 patients (54%) with a cohort median of 1.05 LEVs/mL (range 0–163.95; mean 11.60). A positive correlation was observed between CK|Vim LEVs and CK|Vim|CD45/CD31 LEVs (spearman coefficient = 0.47, *p*-value = 0.001). Both of these LEV populations were detected at a significantly higher level in BCa patient samples than ND samples (*p*-value < 0.0001 for both). The observed LEVs represent additional tumor heterogeneity and a new potential analyte to monitor disease status.

The detection of LEVs was not associated with the detection of epi.CTCs or mes.CTCs. We observed a negative correlation between Vim|CD45/CD31 cells and CK only LEVs (spearman coefficient = −0.39, *p*-value = 0.005). Additionally, a negative correlation was found between CK|Vim LEVs and DAPI only rare cells (spearman coefficient = −0.28, *p*-value = 0.05).

### 3.4. Keck Cohort with Clinical Data

Correlation analysis was used to determine the relationship between the various liquid biopsy analytes and the clinical/demographics metrics collected for the Keck subset of patients (*n* = 22). Here, we report only the significant correlations, whereas a complete table of all comparisons can be found in the Figure S1. A negative correlation was detected between BMI and the Vim only cells/mL (spearman coefficient = −0.41, *p*-value = 0.05), as well as age and the DAPI only cells/mL (spearman coefficient = −0.59, *p*-value < 0.001). WBC count correlated with CK|CD45/CD31 cells/mL (spearman coefficient = 0.47, *p*-value = 0.02) and CK|Vim|CD45/CD31 cells/mL (spearman coefficient = 0.46, *p*-value = 0.03). Platelet count at the time of sample collection correlated with total rare events/mL (spearman coefficient = 0.57, *p*-value < 0.001), total CK expressing cells/mL (spearman coefficient = 0.47, *p*-value = 0.02), mes.CTCs/mL (spearman coefficient = 0.48, *p*-value = 0.02), total LEVs/mL (spearman coefficient 0.61, *p*-value < 0.001), and CK only LEVs/mL (spearman coefficient = 0.63, *p*-value < 0.001). Creatinine blood measurements correlated with CK only LEVs/mL (spearman coefficient = 0.43, *p*-value = 0.04).

Clinical T stage was negatively correlated with CK|CD45/CD31 LEVs/mL (spearman coefficient = −0.62, *p*-value < 0.001). Pathological T stage was negatively correlated with total rare events/mL (spearman coefficient = −0.50, *p*-value = 0.01) and total rare cells/mL (spearman coefficient = −0.53, *p*-value = 0.01). Those patients with Tis had significantly more rare cells/mL than those patients with T3a pathological staging (Wilcoxon = −2.12, *p*-value = 0.03). The significance of the other channel-type rare cells have yet to be determined. Additionally, patients with Tis had a significantly greater CK only LEVs/mL than patients with T3a pathological staging (Wilcoxon = −2.12, *p*-value = 0.03). This suggests that LEVs could be an analyte for early disease.

Total cells/mL, total LEVs/mL, and CK+ LEVs/mL negatively correlated with recurrence (spearman coefficients = −0.44, −0.42, −0.42, respectively; *p*-value < 0.05). The potential for recurrence is low as this prospective study had a median follow-up time since surgery of nine months (range: 6–17) and additional time is warranted for progression/survival data to mature.

### 3.5. Patient Level Classification Modeling

Statistical tests and predictive modeling were used to discern the BCa population from NDs. According to the Wilcoxon rank sum test, the counts/mL detected in NDs belong to different populations than the corresponding samples of BCa for multiple rare event classifications and groups. According to the classification models, the BCa patients and NDs contained distinct cell populations that allowed for stratification, as evidenced by their overall accuracies. The RF, SVM, and NB architectures had average accuracies across their five respective models of 89% ± 9.7%, 87% ± 9.8%, and 83% ± 11.2%, respectively. This corresponds to incorrectly predicting 11 (BCa = 5, ND = 6), 13 (BCa = 8, ND = 5), and 17 (BCa = 12, ND = 5) individuals across each of the models. When looking at the receiver operating characteristic (ROC) curves, the RF yielded an average AUC of 0.94 ± 0.09, as compared to 0.91 ± 0.07 for SVM and 0.90 ± 0.13 for NB. Among the three architectures tested, the RF achieved the highest sensitivity of all models (84% ± 18%), but the lowest specificity (90% ± 9%). Comparatively, the SVM and NB had sensitivities of 79% ± 17% and 70% ± 25% and specificities of 93% ± 10% and 92% ± 10%, respectively.

**Figure 4:**
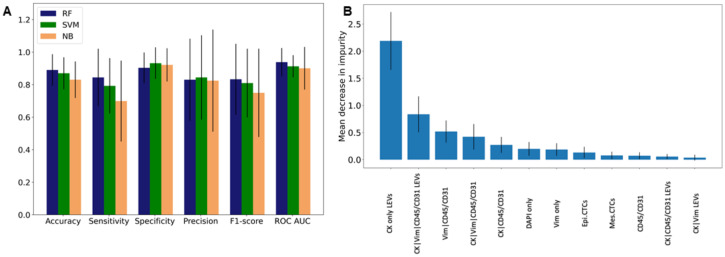
Patient level classification model using liquid biopsy data. Model statistics for (**A**) NB, SVM, and RF. (**B**) Feature importance from RF.

For the RF, the top three most important events for discerning BCa from ND were CK only LEVs, CK|Vim|CD45/CD31 LEVs, and Vim|CD45/CD31 cells (See Figure 4). In fact, six of the top seven events are all statistically different across the two groups, which intuitively makes sense. It is important to note, however, that the most important event, CK only LEVs, is approximately 2.6 times and 4.2 times as important as the following two, respectively. This clearly indicates and supports our previous findings concerning the distinct differences between BCa and ND liquid biopsies.

## 4. Discussion

We have detected liquid biopsy analytes unique to patients diagnosed with BCa prior to cystectomy. More precise clinical diagnostic tools are warranted in the context of BCa to predict response to therapy and monitor minimum residual disease to minimize metastatic progression. This study documents several important findings for liquid biopsy analysis for patients with BCa undergoing cystectomy: (i) CTCs and LEVs are detected in the PB, (ii) there is a high heterogeneity of CTCs, and (iii) liquid biopsy analytes correlate with clinical data elements. The liquid biopsy is a useful non-invasive tool for the discovery of cancer related biomarkers to represent the complex process of tumorigenesis. Our findings suggest that CTC and LEV analysis from the liquid biopsy should be further investigated as an inclusion in BCa patient management.

In summary, our study found that rare cells can be detected in BCa PB samples (median 74.61 cells/mL) as well as ND samples (median 34.46 cells/mL). When specifically considering CK-positive cells, BCa samples presented a median of 27.59 cells/mL while ND samples presented a median of 12.90 cells/mL. This study also found that LEVs can be detected in BCa samples, at a significantly higher count than in ND samples (median 30.91 vs. 3.34 LEVs/mL). Across all BCa samples, both epi.CTCs and mes.CTCs were observed in only 34% and 40% patients, respectively. However, other candidate CTCs were detected at higher frequencies, including CK|CD45/CD31 (median 1.44 cells/mL) and CK|Vim|CD45/CD31 (median 23.19 cells/mL). Additionally, our study found that multiple liquid biopsy analytes both positively and negatively correlated with clinical data metrics, including clinical and pathological T stage, as well as recurrence. For example, patients with Tis disease had significantly more rare cells and CK only LEVs than those with T3a disease.

There are several methods to detect bladder cancer, some more technically challenging and maintaining invasive requirements for the procedure, but different methods have varying degrees of accuracy which depends on the method’s sensitivity and specificity. By having a foundational understanding of the interpretation of sensitivity and specificity, healthcare providers will understand outputs from current and new diagnostic assessments, aiding in decision-making and ultimately improving healthcare for patients. Cystoscopy is invasive and uncomfortable for patients due to the technical requirements of the procedureis still the most accurate diagnosis method for BCa (sensitivity 68–100%, specificity 57–97% [29]. Urine cytology is a non-invasive liquid biopsy approach, and when high-grade tumors are considered, the sensitivity is high (84%), but the sensitivity decreases to 16% in NMIBC, precluding its use in the detection of low-grade lesions [30]. Here, we show that in a mixed cohort (NMIBC and MIBC), applying classification models using liquid biopsy data, we achieved an average sensitivity of 78% and specificity of 92% for the identification of BCa patient samples. We set out to use the liquid biopsy for detection of subclinical metastasis prior to surgical resection. While this remains our primary goal, the data also support the general consideration of the liquid biopsy for the screening and diagnostic work-up of BCa.

The liquid biopsy might be an indicator of early disease dissemination with micrometastases, and assessment prior to cystectomy is therefore crucial. CellSearch^®^ CTCs were detectable in 8/44 NMIBC patients at diagnosis (18%) in which the presence of CTCs was associated with a shorter time to first recurrence [31]. Using the HDSCA3.0 workflow, we detected epi.CTCs in 38% and mes.CTCs in 46% of BCa patients presented here. However, there was no statistical difference between the same type of cells detected in the ND samples. The detection of CTCs prior to cystectomy in BCa patients has been shown to serve as evidence of progressing disease which may predict the appearance of a macroscopic lesion in a longer-term period. Therefore, patients with low CTC counts before cystectomy are hypothesized to have a low risk of recurrence and are thus good candidates for cystectomy [11]. Additional time is needed to monitor the progress of the BCa patients in this study and determine if our hypothesis is correct.

A heterogeneous population of rare cells was observed in the PB of BCa patients prior to cystectomy. Here, we identified eight categories of rare cells based on the expression of four biomarkers (CK, Vim, CD45/CD31), but further cellular stratification could be conducted using morphometric parameters as these categories include a mixture of cell types as seen by morphological analysis. Since the total rare cell count/mL was correlated with pathological T staging (spearman coefficient = −0.53, *p*-value = 0.01), we conclude that the rare cells detected are indeed related to disease status. This is evidence for the circulation of multiple CTC populations and other rare cells, possibly from the tumor microenvironment (TME), as measures of tumor burden and disease state. Furthermore, since the HDSCA3.0 workflow detects rare cells beyond the epi.CTCs, the negative association between total rare cells/mL and tumor stage may be driven by the high frequency of cells other than CTCs that may represent the tumor microenvironment (TME). We hypothesize that rare cell populations expressing Vim|CD45/CD31 include circulating endothelial cells (CECs). In a prior publication, we showed that CECs (CD138|von Willebrand Factor positive, CD45 negative) in PB samples were morphologically distinct from the surrounding WBCs, and CEC count was significantly higher in myocardial infarction patients than that of the healthy control [32]. The presence of CECs in the PB may be a novel way to assess vascular function in BCa patients, potentially as markers of altered vascular integrity or even direct contributors to tumor formation (i.e., angiogenesis). Further characterization is warranted to understand the biological significance of each channel-type cellular population, but this study highlights the promise of the liquid biopsy for early risk stratification of BCa patients, prediction of treatment response, and early detection of metastatic relapse.

Here, we show that circulating LEVs have been detected in an enrichment-free liquid biopsy approach, representing a promising new analyte for BCa care. Tumor heterogeneity is further seen in the four different LEV categories detected. The results presented here demonstrated a statistically higher overall presence of tumor-associated LEVs in BCa patients prior to cystectomy compared to the NDs (median 30.91 LEVs/mL vs. 3.34 LEVs/mL, respectively), most likely due to the presence of the primary tumor. Exosomes contain a number of analytes (nucleic acids, proteins, and metabolites) which strongly reflect the parental cell properties, making them a promising alternative to CTCs or circulating tumor DNA (ctDNA) as biomarkers of disease. In a study of extracellular vesicles (EV; size 30–200 nm) detected from urine, BCa patients had higher concentration of EVs in the urine when compared with healthy controls, with a sensitivity of 81.3% and a specificity of 90.0% in the discrimination of BCa patients against healthy controls [33]. This supports the utility of LEVs in the diagnostic work-up for BCa clinical care. In prostate cancer, LEVs detected in the PB using the same workflow were 1.9 times as frequent as CTCs and shared a similar protein signature [20]. Here, we show that LEVs are associated with BCa tumorigenesis and may be useful diagnostic and prognostic biomarkers. Further characterization of the LEVs detected here will validate their neoplastic origin and association with the BCa disease state.

The molecular characterization of the rare events detected in this study will elucidate their potential role in BCa tumorigenesis. Molecular profiling through genomic and proteomic analysis of a patient’s liquid biopsy will have value in enabling the discovery of novel drivers of growth and metastasis that help direct individual treatment or identify potential new treatment targets. Using the HDSCA workflow, we have the unique opportunity for a comprehensive analysis of the liquid biopsy [13,14,21,34,35,36,37]. Previous studies have used single-cell sequencing and targeted multiplexed proteomic analysis to characterize both circulating rare and common cells detected by the HDSCA workflow in a variety of clinical scenarios [13,14,21,34,35,38]. Additionally, cfDNA genomic analysis is possible for a more comprehensive view of the liquid biopsy. Multiple prior studies indicate that ctDNA is detectable in plasma of BCa patients, and high levels of ctDNA are associated with progression and metastatic disease [39,40,41,42]. Chalfin et al., show that CTC and ctDNA provide complementary information in urothelial carcinomas [43]. The ability to characterize tumor heterogeneity using a single platform with comprehensive single-cell DNA, single-cell multiplexed targeted proteomics, and cfDNA analysis could provide precision diagnostics from the time of initial diagnosis for patients with BCa. Future research aims to establish evidence towards the clinical utility of the liquid biopsy in BCa to predict metastatic relapse post cystectomy and enable clinical intervention to lead to improved outcomes.

## 5. Conclusions

This study establishes evidence for the clinical utility of the liquid biopsy in BCa with the future goal of predicting metastatic relapse post-cystectomy and enabling clinical intervention that can lead to improved outcomes. Here, we show the identification of rare cells and LEV frequencies unique to BCa patients, with distinct populations within and across patients underscoring the heterogeneity of liquid biopsy profiles. Further, the high specificity and sensitivity metrics of the prediction models demonstrate the stratification of BCa patients from ND using this methodology. While further investigation is needed to elucidate the predictive power of these analytes with respect to recurrence, the findings from this study show the liquid biopsy as a promising clinical tool for early-stage BCa patients.

## Figures and Tables

**Table 1:** Clinical demographics for Keck subset of patients.

Variable	Category	Value
Age		71.4 (53.4–86.1)
BMI		24.9 (21.2–36.9)
Hgb		11.1 (5.1–15.0)
HCT		34.2 (18.3–46.3)
WBC		7.6 (4.8–20.4)
Platelets		201.5 (57–387)
BUN		22.5 (13–70)
Creatinine		1.2 (0.5–3.1)
Race	Caucasian	20
	Asian	2
Gender	Male	18
	Female	4
Smoker	Previous	14
	Current	4
	Never	4
Neoadjuvant Chemo	Yes	10
	No	12
Surgical Procedure	Anterior Exenteration	1
	Radical Cystectomy	4
	Robotic Radical Cystectimy	17
Urinary Diversion	Studer	9
	Ileal Conduit	11
	Indiana Pouch	2
Pure Urothelial (CS/PS)		7/4
Predominant Histology (CS/PS)	No Tumor	2/9
	Urothelial	17/11
	Other	3/1
	Plasmacytoid	0/1
Squamous (CS/PS)	Absent	16/12
	Present	2/1
	NA	4/9
Glandular (CS/PS)	Absent	16/12
	Present	2/1
	NA	4/9
Neuro (CS/PS)	Absent	18/12
	Present	1/1
	NA	3/9
Subgroup (CS/PS)	OC	16/15
	EV	4/3
	N+	2/4
T Stage (CS/PS)	T0	2/9
	Ta	2/0
	Tis	1/4
	T1	1/2
	T2a	11/0
	T2b	0/1
	T3a	0/3
	T3b	2/1
	T4a	3/2
N Stage (CS/PS)	NX	2/0
	N0	19/18
	N2	1/4

Abbreviations: CS, clinical staging; PS, pathological staging; OC, organ confined; EV, extravesical; N+, node positive; BMI, body mass index; Hgb, hemoglobin; HCT, hematocrit; WBC, white blood cell; BUN, blood urea nitrogen.

## Data Availability

All data discussed in this manuscript are included in the main manuscript text. The images of the single cells and LEVs are available through the BloodPAC Data Commons Accession ID “BPDC000120”.

## Supplementary Materials

The following are available online at https://www.mdpi.com/article/10.3390/cancers14030758/s1, Figure S1: Site specific liquid biopsy data (Keck; *n* = 25, JHH; *n* = 13, UCSD; *n* = 9, LAC; *n* = 3). (A) Bar plot of average counts per patient for each classification and across sites. (B) Logarithmic box plot of counts per patient for each classification across sites.
