# Supplementary material for: Characterization of Cellular and Acellular Analytes from Pre-Cystectomy Liquid Biopsies in Patients Newly Diagnosed with Primary Bladder Cancer
Source: Cancers (Basel). 2022 Feb 1;14(3):758. doi: 10.3390/cancers14030758 (PMC8833768; doi:10.3390/cancers14030758)
Supplement: Supplementary file 1 [file cancers-14-00758-s001.zip › cancers-1543508-supplementary.pdf]

cancers

Supplementary Materials

Characterization of Cellular and Acellular Analytes from Pre-Cystectomy Liquid Biopsies in Patients Newly Diagnosed with Primary Bladder Cancer

Stephanie N. Shishido, Salmaan Sayeed, George Courcoubetis, Hooman Djaladat, Gus Miranda, Kenneth J. Pienta, Jorge Nieva, Donna E. Hansel, Mihir Desai, Inderbir S. Gill, Peter Kuhn and Jeremy Mason

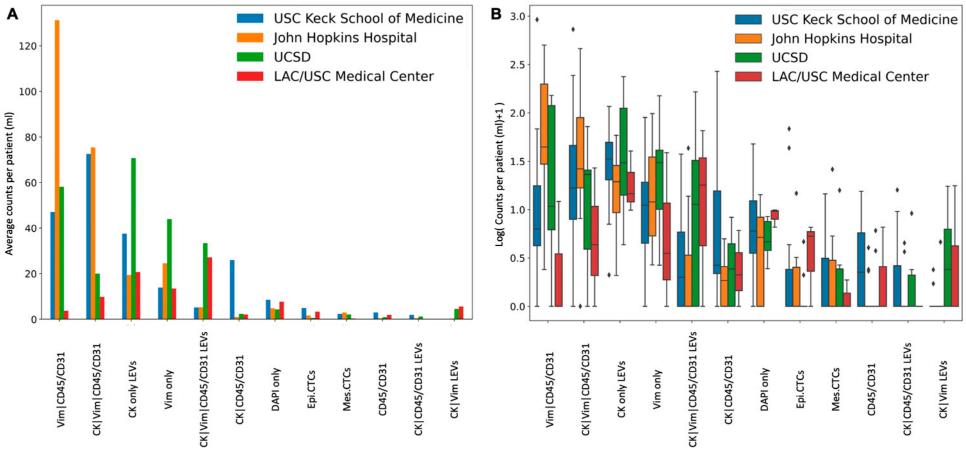

Figure S1. Site specific liquid biopsy data (Keck; n = 25, JHH; n = 13, UCSD; n = 9, LAC; n = 3). (A) Bar plot of average counts per patient for each classification and across sites. (B) Logarithmic box plot of counts per patient for each classification across sites.
